# Improved clinical communication OSCE scores after simulation-based training: Results of a comparative study

**DOI:** 10.1371/journal.pone.0238542

**Published:** 2020-09-04

**Authors:** Alexandre Nuzzo, Alexy Tran-Dinh, Marie Courbebaisse, Hugo Peyre, Patrick Plaisance, Alexandre Matet, Brigitte Ranque, Albert Faye, Victoire de Lastours, on behalf of the University of Paris OSCE and SBT groups

**Affiliations:** 1 Université de Paris, Faculté de Médecine Paris-Diderot, Paris, France; 2 Université de Paris, Faculté de Médecine Paris-Descartes, Paris, France; University of Newcastle, AUSTRALIA

## Abstract

**Objectives:**

Simulation-based training (SBT) is increasingly used to teach clinical patient-doctor communication skills (CS) to medical students. However, the long-lasting impact of this training has been poorly studied.

**Methods:**

In this observational study we included all fourth-year undergraduate medical students from a French medical school who undertook a CS objective structured clinical examination (OSCE) and who answered a post-examination survey. OSCE scores and students’ feedback were compared by whether students had received a specific CS-SBT or not 12 months prior to the OSCE.

**Results:**

A total of 173 students were included in the study. Of them, 97 (56%) had followed the CS-SBT before the OSCE. Students who had undergone CS-SBT had significantly higher CS-OSCE scores in the multivariate analysis compared to untrained students (mean score 7.5/10 ±1.1 vs. 7.0/10 ±1.6, respectively, Cohen’s *d* = 0.4, *p*<0.01). They also tended to experience less nervousness during the OSCE (*p* = 0.09) and increased motivation to further train in “real-life” internships (*p* = 0.08). However, they overall expressed a general lack of CS in therapeutic patient education, delivering bad news, and disclosing medical errors.

**Conclusions:**

Fourth-year medical students who benefited from a CS-SBT 12 months before examination displayed higher CS-OSCE scores than their counterparts.

**Practice implications:**

These results support the early introduction of practical training to improve communication skills in undergraduate medical curricula. Studies are required to assess the sustainability of this improvement over time and its effect on further real doctor-patient communication.

## Introduction

Effective doctor-patient communication is an essential physician’s skill. In recent years, the medical literature has addressed the importance of improving physician‐patient interaction [1]. Studies that have analyzed the role of physicians’ communication and their ability to adapt to the patient’s personality have shown improved patient satisfaction and treatment outcomes among physicians trained in communication skills (CS) [2].

Simulation-based training (SBT) could be a useful tool to improve CS [3–5]. Several studies have reported improvements of CS by SBT and when taught early in undergraduate medical curricula [6–10]. However, evidence remains scarce about the potential lasting effect of SBT [4].

Developed in 1975 by Harden and colleagues, the objective structure clinical examination (OSCE) is now widely used to assess clinical competence in medical education and provides a unique tool for evaluating the impact of different training programs [11]. In particular, OSCE is preferred over paper-and-pencil tests of knowledge to assess clinical CS using interactions with standardized patients.

In France, the implementation of CS simulation-based training (CS-SBT) is novel and many students still do not have access to this training. In our university, CS-SBT has been offered from 2017 as an optional course for third-year students. The aim of this observational study was to compare undergraduate medical students OSCE scores according to whether they had received CS-SBT 12 months before or not.

## Methods

### Study design

**Fig 1:**
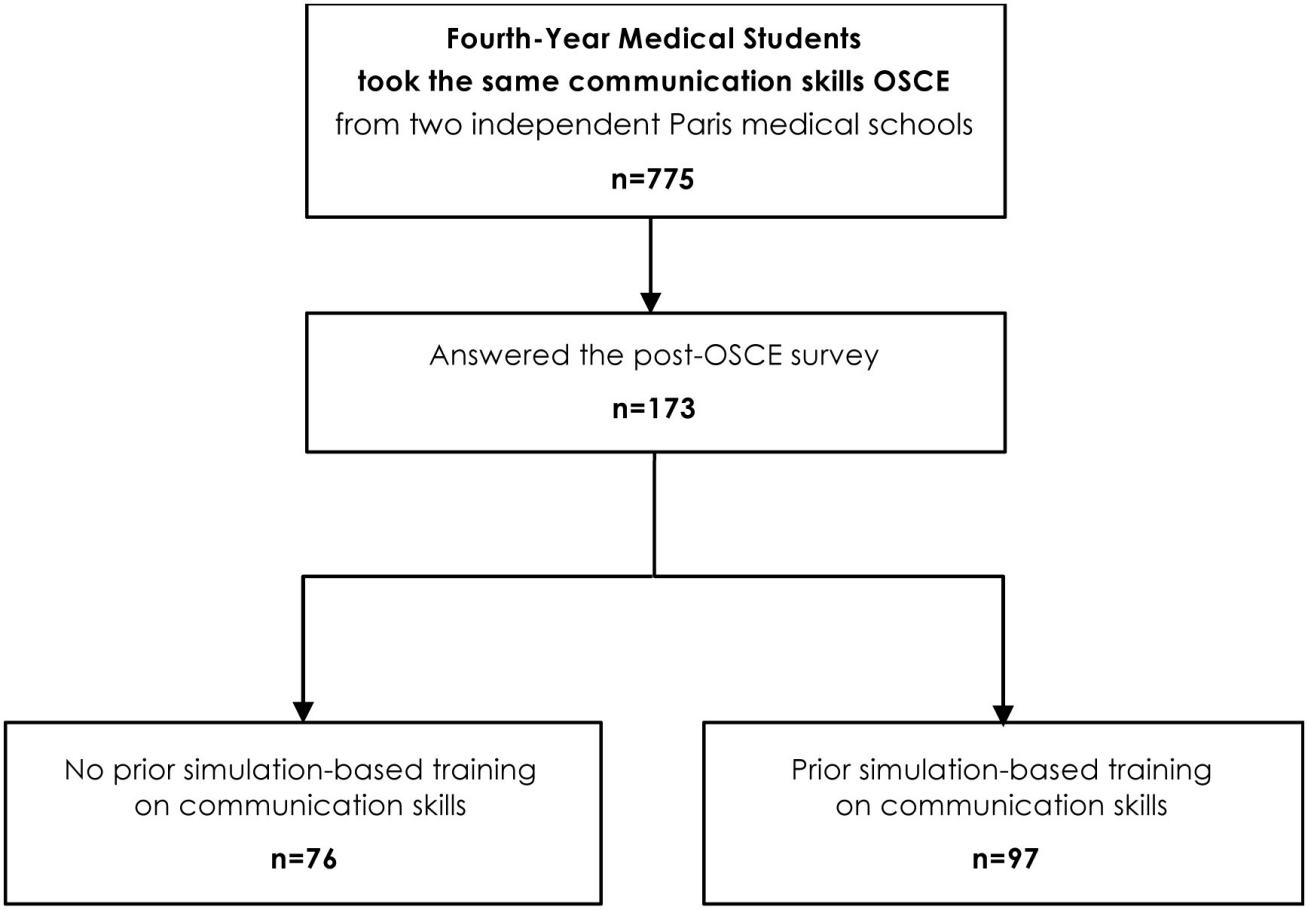
Flowchart of the communication skills OSCE participants. Abbreviations: OSCE: objective structured clinical examination.

We conducted an observational cohort study at the University of Paris medical school, which has two main campuses (Paris Nord and Paris Centre). All fourth-year undergraduate medical students who undertook an OSCE using standardized patients as part as their mandatory training in May 2019 and who answered to the anonymous post-examination survey were included (flowchart, Fig 1). The study was approved by the education council and review board of University of Paris and informed consent was waived (data analyzed anonymously).

### Clinical communication skills educational programs

During the 3^rd^ year of their training, medical students had been offered optional courses on CS:

- One of the following two SBT programs: SBT offered by Paris Centre campus including role-playing for 1.5 hours and consultation with standardized simulated patients for 1 hour, or SBT offered by Paris Nord campus delivered in a dedicated health simulation center and consisting of a 3-hour session including two clinical scenarios of 15min with simulated patients, two observing students and one supervising teacher, with personalized and global feedback. Briefly, students played their role of medical students meeting a patient for the first time and had to take his medical history. A senior medical doctor role-played the patient. Both tutors and other students had the opportunity to evaluate the relational dynamics in the role-play by observing their interaction through a one-way screen. After the role-play, they debriefed the content and way of communicating in a structured way.
- A theoretical CS training by conventional lectures (2 hours).

No other CS educational programs took place between the CS-SBT followed during their 3^rd^ year and the OSCE undertook during their 4^th^ year of medical studies, apart from the continuous bed-side training received during their internships at teaching hospitals.

### OSCE setting and case scenario

The OSCE included four 7-minute stations. For each OSCE station, two faculty examiners were assigned; one role-played the standardized patient, and the other one was the evaluator. All faculty examiners had undergone a role-playing and evaluation training. This training included 1) an on-site course about the OSCE process and their role as examiners and simulated patient and 2) an online video showing how the clinical scenario should be played to standardize the patient’s part.

Briefly, the CS-OSCE station case scenario had the students manage the patient’s stress on the day before a scheduled cholecystectomy (S1 and S2 Data). Eighty-eight faculty examiners (44 standardized patients and 44 evaluators) scored the medical students’ CS through this specific OSCE station on the doctor-patient relationship, following a standardized rating scale derived from the Calgary Cambridge modified guide (Table 1) [12]. This standardized rating scale had been pre-tested by medical teachers and residents of both universities to ensure the feasibility, reliability and reproducibility of the OSCE station’s scoring system.

**Table 1:** Rating scale of the communication skills objective structured clinical examination station.

Tasks and aims	Acquired	Score
Introduces himself with his name		0.5
Introduces himself with his function		0.5
Check that the antecedent of allergy has been mentioned with the doctors (surgeon, anesthesiologist)		1
Reassure the patient that this allergy will be taken into account and that she will not receive penicillin during her hospitalization		1
Check for other allergies		1
Avoid recurrences of biliary colic		0.5
Avoid acute complications (infection, acute pancreatitis)		0.5
Empathy, listening		1
Avoid medical jargon, or take the time to explain it		1
Ask the patient if he understood the explanations given		1
Ask the patient if he has any other questions		1
General attitude (posture, smile, look at the patient in the eyes)		1
**Total score / 10**		10

### Outcomes

The primary outcome was the CS-OSCE station scores, as measured by a standardized rating scale (total score = 10) derived from the Calgary Cambridge modified guide (Table 1). Secondary outcome measures included their experience feedback based on an anonymous online survey. The post-examination survey collected their feedback about OSCE training, organization, expectations, and needs related to CS teaching. Feedback items were rated by students via a 5-point Likert scale. For each item, mean Likert-scale score / 10 points was calculated as follows: Strong Disagreement = 0, Disagreement = 2.5, Neutral = 5, Agreement = 7.5, Strong Agreement = 10 points. Students, CS-SBT teachers and OSCE examiners were unaware of the study and blinded to the outcomes measured.

### Statistical analysis

Qualitative data were reported as the number of patients (percentage of patients) and were compared using either the Pearson χ2 test or the Fisher exact test, depending on the sample size. Continuous and ordinal data such as OSCE scores and Likert scale ratings were reported as means (standard deviation) and analyzed with the Mann-Whitney U test. Missing data were not analyzed or estimated. Students’ CS-OSCE station scores and feedback (Likert scale ratings) were compared according to whether they had received prior SBT or not. An adjustment was performed using an analysis of covariance (ANCOVA) model using CS-OSCE station scores as the dependent variable, prior CS-SBT as the independent variables, and the following covariates: gender, the medical school of origin and attendance to prior conventional lectures. All tests were two-sided. A p-value < 0.05 was considered to be significant. All analyses were performed using the Statistical Package for the Social Sciences (SPSS) for Mac OSX software (version 23.0, Chicago, Illinois, USA).

## Results

### Participants

In May 2019, a total of 775 medical students (4^th^-Year of Medical School– 379 from Paris Centre campus, and 396 from Paris Nord campus) took the same 7-minute OSCE station focused on doctor-patient communication skills. A total of 173/775 medical students (22.3%) answered the post-examination online survey– 107 (61.8%) from the Paris Centre campus and 66 (38.2%) from the Paris Nord campus. It constituted the final cohort of students analyzed in this work (see flowchart, Fig 1). The survey respondents’ average CS-OSCE station score was 7.3/10 ±1.4 (*vs*. 7.1/10 ±1.5 for the overall 775 students who took the OSCE, one-sample t-test *p* = 0.04). The baseline characteristics of the 173 participants are summarized in Table 2.

**Table 2:** Baseline characteristics of the 173 fourth-year medical students.

	Students n = 173 (%)
Female	121 (70)
Paris Centre campus	107 (62)
Paris Nord campus	66 (38)
**Communication skills simulation-based training**	**97 (56)**
*Simulation-based training only*	*27 (16)*
*Simulated-based training and lectures*	*70 (40)*
**No communication skills simulation-based training**	**76 (44)**
*No training*	*13 (8)*
*Lectures only*	*63 (36)*

### Communication skills simulation-based training (CS-SBT)

**Fig 2:**
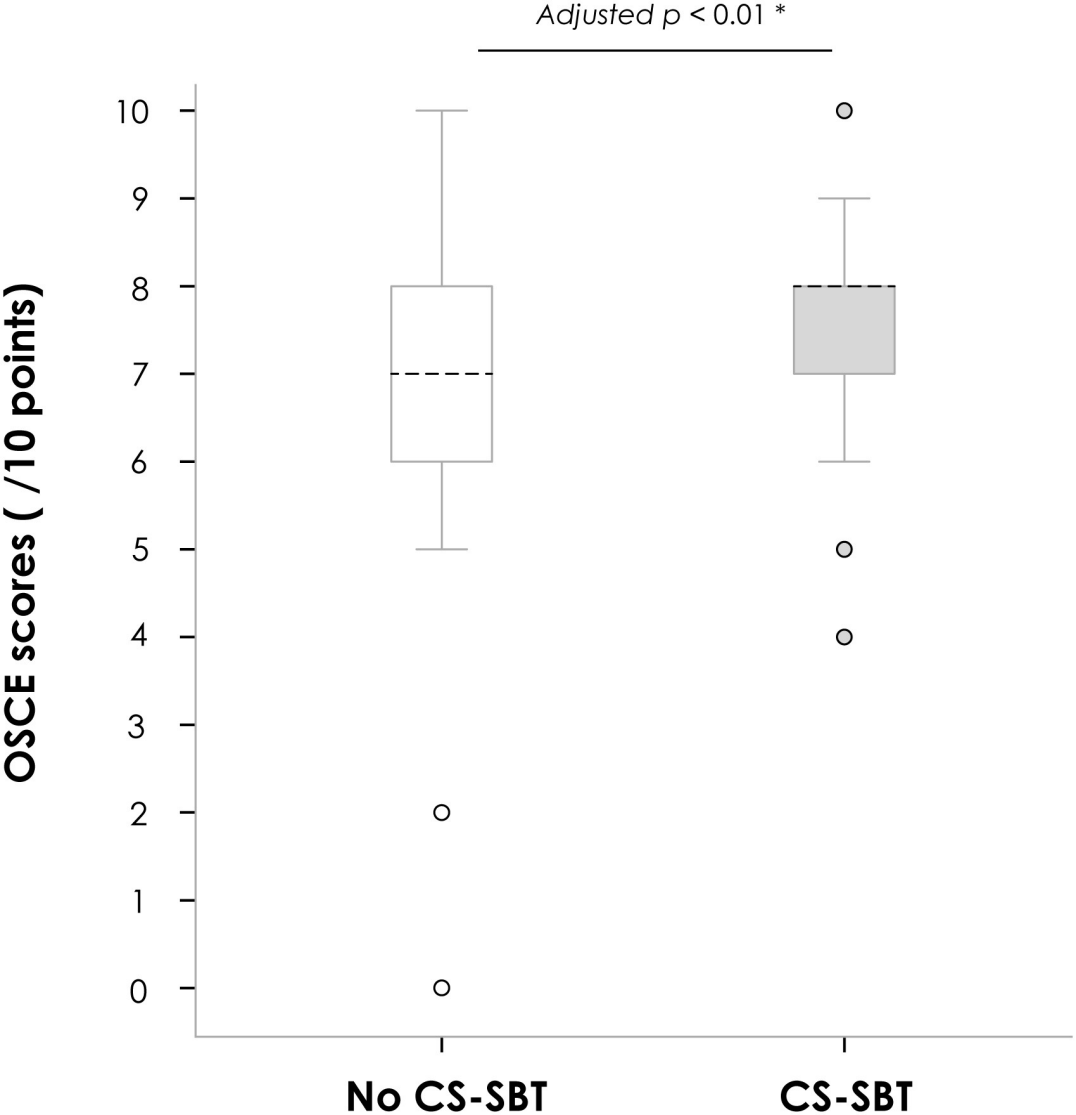
Communication skills (CS) OSCE station scores according to the CS-simulation-based training (SBT) status. Abbreviations: OSCE: objective structured clinical examination; CS-SBT: communication skills simulation-based training. The horizontal black dotted line in the boxes represent the median, and the bottom and top of the boxes the 25th and 75th percentiles, respectively. I bars represent the upper adjacent value (75th percentile plus 1.5 times the interquartile range) and the lower adjacent value (corresponding formula below the 25th percentile), and the dots outliers. *Adjusted for gender, medical school campus and prior attendance at conventional lectures (ANCOVA model).

Of the 173 students included, 97 (56%) had received specific CS-SBT 12 months before the OSCE (Table 2). The seventy-six students (44%) who did not follow any CS-SBT included 63 (36%) who had attended the conventional lectures only and 13 (8%) with no prior training at all (Table 2). Students who had undergone CS-SBT had significantly higher CS-OSCE station scores independently from gender, medical school campus, and previous attendance of conventional lectures (mean 7.5/10 ±1.1 vs. 7.0/10 ±1.6 in non-trained students, Cohen’s *d* = 0.4, adjusted *p*<0.01) (Fig 2). No significant difference was found between students with no prior training vs. conventional lectures only (*p* = 0.11) or between students with lectures + CS-SBT and CS-SBT only (*p* = 0.54) (S3 Data). Furthermore, CS-SBT students tended to be less nervous during the OSCE (*p* = 0.09), requested more feedback from the examiners, and showed increased motivation to further train CS in “real-life” internships (*p* = 0.08) (Table 3).

**Table 3:** Students communication skills OSCE station scores and feedback according to their simulation-based training status.

	No CS-SBT n = 76	CS-SBT n = 97	*p-value*
**Female**, n (%)	49 (65)	72 (74)	0.17
**Paris-Centre campus** (vs Paris-Nord campus, n, %)	53 (70)	54 (56)	0.06
**Conventional lectures**, n (%)	63 (83)	70 (72)	0.10
**OSCE station scores**, mean /10 ±SD	7.0 ±1.6	7.5 ±1.1	0.04
**Opinion on the OSCE station**, mean rating/10 ±SD ^1^			
Difficulty	4.3 ±2.5	4.1 ±2.0	0.73
Nervousness	5.9 ±2.5	5.1 ±3.0	0.09
Simulated-patient good acting	6.4 ±2.6	6.6 ±2.8	0.51
Lack of a personnal feedback from the examiner	8.8 ±2.2	9.3 ±1.5	0.05
Motivates to train in real-life internships	7.1 ±3.0	8.0 ±2.5	0.08
**Opinion on the SBT program**, mean rating/10 ±SD ^1^			
It is more efficient than lectures	7.7 ±2.1	9.0 ±1.7	< 0.01
It can decrease per-OSCE nervousness	6.3 ±2.5	6.4 ±2.9	0.64
It can improve OSCE scores	7.0 ±2.3	6.6 ±2.8	0.59
It is adapted to OSCE needs	7.4 ±2.0	6.8 ±2.9	0.64
More training sessions are needed	7.3 ±2.8	7.4 ±2.6	0.70
It could improve clinical skills as a senior doctor	8.1 ±2.4	8.6 ±1.9	0.32
**Self-assessment of their communication skills**, mean rating/10 ±SD ^1^			
Listening with empathy	8.3 ±1.6	8.6 ±1.5	0.16
Avoiding medical jargon	7.4 ±2.0	7.4 ±2.2	0.87
Therapeutic patient education	5.7 ±2.2	6.0 ±2.4	0.30
Delivering bad news	3.5 ±2.6	3.5 ±2.9	0.97
Disclosing a medical error	2.5 ±2.4	2.8 ±2.6	0.48

Abbreviations: OSCE: objective structured clinical examination; SD: standard deviation; CS-SBT: communication skills simulation-based training

^1^ Mean Likert-scale score / 10 ±SD), on a 5-point scale score calculated as follows: Strongly Disagree = 0, Disagree = 2.5, Neutral = 5, Agree = 7.5, Strongly Agree = 10.

### Student’s feedback on CS-OSCE and SBT

**Fig 3:**
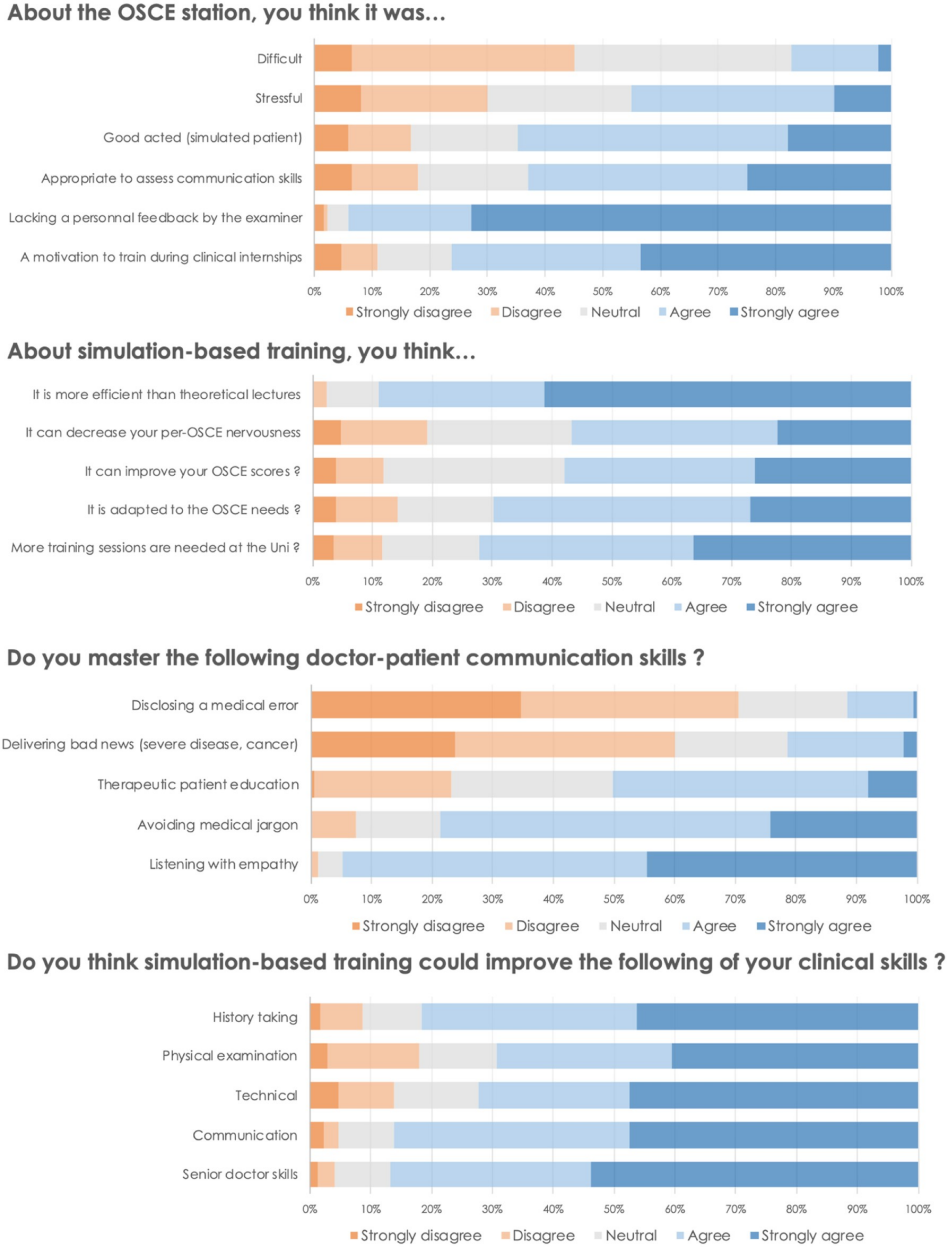
Overview of the students’ answers (Likert-scale ratings) to the post-OSCE survey (staggered bars).

Students’ survey answers are summarized in Fig 3 and Table 3. The overall mean score of the clinical scenario difficulty was 4.3/10 ±2.3, and the rating of the nervousness experience during OSCE was 5.4/10 ±2.8. The statement that SBT may be more efficient than conventional lectures on teaching CS and that SBT motivates the students to train more during their internships reached mean scores of 8.7/10 ±1.9 and 8.2/10 ±2.1, respectively. The feeling that SBT could improve their global clinical skills as a future resident or senior doctor achieved a mean rating of 8.4±2.2 /10. The CS where students felt confident included listening and avoiding jargon (respectively were rated 8.5/10 ±1.6 and 7.4/10 ±2.1) whereas therapeutic education, bad news delivery, and medical error disclosure were rated 5.9/10 ±2.3, 3.5/10 ±2.8 and 2.7/10 ±2.5, respectively. Correlation studies showed a moderate inverse association between students’ nervousness and OSCE scores (Spearman r = -0.35, *p*<0.001). A positive correlation was found between the quality of acting from the role-played standardized patient and OSCE scores (r = 0.28, *p*<0.001) (correlation table, S4 Data).

## Discussion

In this comparative study of 173 fourth-year medical students from a French medical school, CS-OSCE station scores were significantly higher in students who participated in a simulation-based training program (SBT) one-year before the OSCE. This study is one of the first evaluating CS-SBT training programs delivered early in the undergraduate medical curriculum, and assessing their lasting impact 12 months after with a comparative group [4]. This training evaluation corresponds to a Kirkpatrick level 2b as the OSCE examined how well students could practically apply what they learned from the training received 12 months before [13]. Mean OSCE station scores were 0.5 points higher (corresponding to a median 1 point higher, on a total of 10 points) in students who had undergone prior CS-SBT, independently from gender, medical school, and attendance to conventional lectures (adjusted *p*<0.01). CS-SBT students tended to experience less nervousness and showed increased motivation to receive feedback from examiners and to further train in “real-life” internships. Additionally, as the CS-SBT training was offered one year before the OSCE, our results suggest that this teaching approach has a lasting benefit. On the other hand, we did not find any significant difference in OSCE scores from students who had received conventional lectures only compared to those who had not had any specific training. Also, no difference was found in students who had traditional lectures + CS-SBT compared to CS-SBT only. The latter result would suggest a lower efficacy of conventional lectures on CS, although the comparison was underpowered. However, student’s positive feedback claimed for more training as they believe it could enhance the whole array of their clinical expertise, including their future skills as a resident and senior doctor. Especially, irrespective of whether they had prior training or not, students rated poorly their competence in delivering therapeutic education, bad news, or medical errors, consistent with previous studies [14]. Therefore, further efforts should be made to improve the teaching and assessment of those particularly challenging clinical skills, which represent paramount needs of patients and their families. Students often claim that teaching could be improved in educational programs to enable them to develop better and maintain their communication skills [15, 16]. Several studies have supported the early introduction of practical training to improve communication skills in undergraduate medical students, which allows them to apply their theoretical knowledge continually and facilitates their learning [4, 15–17]. However, further studies are required to determine the long-term effects of such programs on later professional skills.

Based on our results, the positive effect of CS-SBT may be multimodal, impacting the quality of their CS but also their nervousness, motivation, commitment, and their desire for self-improvement and self-training with real patients during their internships. Indeed, we found an upward trend in nervousness ratings in untrained students (no CS-SBT), inversely correlated with OSCE scores. Nervousness was also inversely correlated with the good-acting of the simulated patient by the faculty examiner, confirming that the authenticity and the standardization of the role-play are critical to ensure students’ equity and avoid OSCE scores biases [18].

Some limitations should be discussed. First, although OSCE were mandatory, CS-SBT was an optional course. Second, only 22% of OSCE participants answered the anonymous post-OSCE survey and were included in the analysis. This may have selected students more motivated by the SBT as a result of specific training needs or higher interest in CS, as we observed that the average OSCE station score of our sample population of 173 students was slightly higher than the entire cohort of 775 students who took the OSCE. However, groups with or without prior SBT were reasonably balanced, and the multivariate analysis controlled for most confounders. Moreover, students and examiners were unaware of the study at the time of CS-SBT and OSCE and blinded to the outcomes measured, which may have limited evaluation biases. Besides, randomised controlled trials of CS training may be difficult to implement while ensuring equity across all the students. Third, the difference in OSCE score between students with and without the SBT was mean 0.5 out of 10 points (median 1 point). Although this result was statistically significant, one may wonder whether this improvement would be noticeable from a patient perspective. Such a level of evidence is, however, rarely achieved in medical education studies. The small but lasting achievement observed in our study remains promising. Finally, although our correction grid included some medical knowledge items, communication skills elements were predominant, and there is no consensus on which correction grid to use to assess clinical communication of medical students in OSCE [19]. Indeed, the validity of the performance scores of a student is fundamentally dependent on the quality of the rating scales in use [20]. Different kinds of rating scales have been developed to assess student’s communication skills performances during an OSCE [21–25]. For this OSCE station, our rating scale was developed from a Calgary Cambridge modified guide [22].

In conclusion, our results support the implementation of a practical SBT program to teach CS to undergraduate students early in the medical curricula and suggest a lasting benefit as measured by a specific OSCE undertaken 12 months later. Further studies are required to investigate the effect of SBT on other challenging communication skills such as breaking bad news and the sustainability of this improvement in future medical practice.

## Supporting information

S1 Data Instruction for students. (DOCX)

S2 Data Instruction for the standardized patient case scenario. (DOCX)

S3 Data Communication skills OSCE station scores according to the training status. (DOCX)

S4 Data Spearman correlations matrix for OSCE scores and covariates. (DOCX)

S1 File (XLSX)

## Abbreviations

CS: communication skills
OSCE: objective structured clinical examination
SBT: simulation-based training
