# Supplementary material for: Improved clinical communication OSCE scores after simulation-based training: Results of a comparative study
Source: PLoS One. 2020 Sep 4;15(9):e0238542. doi: 10.1371/journal.pone.0238542 (PMC7473530; doi:10.1371/journal.pone.0238542)
Supplement: S1 Data — (DOCX) [file pone.0238542.s001.docx]

**Supplemental data 1: Instruction for students**

You are a medical student on an internship in the department of digestive surgery. Mr (Mrs) D. was admitted to hospital the day before scheduled cholecystectomy surgery for biliary colic. The resident surgeon asks you to initiate a medical interview with the patient because the nurse reports a patient who is stressed and has questions to ask doctors.

You will be evaluated on the relevance of the answers given to the patient’s questions and on your general attitude towards his stress. You do not need to perform a physical examination.
