# Supplementary material for: Improved clinical communication OSCE scores after simulation-based training: Results of a comparative study
Source: PLoS One. 2020 Sep 4;15(9):e0238542. doi: 10.1371/journal.pone.0238542 (PMC7473530; doi:10.1371/journal.pone.0238542)
Supplement: S2 Data — (DOCX) [file pone.0238542.s002.docx]

**Supplemental data 2: Instruction for the standardized patient case scenario**

You are Mr (Mrs) D., you are 55 years old, and you are particularly anxious before the surgery. You have questions for doctors.

You have a history of angioedema after taking amoxicillin prescribed by your general practitioner for a rebellious cough last winter. On that occasion, you were hospitalized for two days, and you were terrified. You are now very worried about the risk of a new intake of amoxicillin. You ask the doctor to reassure you.

Besides, you are also very stressed by tomorrow’s surgery, and you wonder whether this surgery is still necessary. Indeed, you have understood very well the technical aspects of the operation, but you wonder about its interest, given that you no longer have acute abdominal pain.
