# Supplementary material for: Improved clinical communication OSCE scores after simulation-based training: Results of a comparative study
Source: PLoS One. 2020 Sep 4;15(9):e0238542. doi: 10.1371/journal.pone.0238542 (PMC7473530; doi:10.1371/journal.pone.0238542)
Supplement: S3 Data — (DOCX) [file pone.0238542.s003.docx]

**Supplemental data 3: Communication skills OSCE station scores according to the training status**

Abbreviations: OSCE: objective structured clinical examination; CS-SBT: communication skills simulation-based training

The horizontal black dotted line in the box represent the median. The top and bottom of the box represent the 75^th^ and 25^th^ percentiles, and the black dotted line in the box represent the median. I bars represent the upper adjacent value (75th percentile plus 1.5 times the interquartile range) and the lower adjacent value (corresponding formula below the 25th percentile), and the dots outliers.

*Adjustment for gender, medical school and prior attendance at conventional lectures (ANCOVA model)
