# Supplementary material for: Improved clinical communication OSCE scores after simulation-based training: Results of a comparative study
Source: PLoS One. 2020 Sep 4;15(9):e0238542. doi: 10.1371/journal.pone.0238542 (PMC7473530; doi:10.1371/journal.pone.0238542)
Supplement: S4 Data — (DOCX) [file pone.0238542.s004.docx]

**Supplemental data 4: Spearman Correlations Matrix for OSCE scores and covariates**
